# Identification of a new hominin bone from Denisova Cave, Siberia using collagen fingerprinting and mitochondrial DNA analysis

**DOI:** 10.1038/srep23559

**Published:** 2016-03-29

**Authors:** Samantha Brown, Thomas Higham, Viviane Slon, Svante Pääbo, Matthias Meyer, Katerina Douka, Fiona Brock, Daniel Comeskey, Noemi Procopio, Michael Shunkov, Anatoly Derevianko, Michael Buckley

**Affiliations:** 1 RLAHA, University of Oxford, OX1 3QY, UK; 2 MPI-EVA, Leipzig, 04103, Germany; 3 Cranfield Forensic Institute, Cranfield University, SN6 8LA, UK; 4 Faculty of Life Sciences, University of Manchester, M13 9PL, UK; 5 Institute of Archeology and Ethnography, Novosibirsk, 630090, Russia

## Abstract

DNA sequencing has revolutionised our understanding of archaic humans during the Middle and Upper Palaeolithic. Unfortunately, while many Palaeolithic sites contain large numbers of bones, the majority of these lack the diagnostic features necessary for traditional morphological identification. As a result the recovery of Pleistocene-age human remains is extremely rare. To circumvent this problem we have applied a method of collagen fingerprinting to more than 2000 fragmented bones from the site of Denisova Cave, Russia, in order to facilitate the discovery of human remains. As a result of our analysis a single hominin bone (Denisova 11) was identified, supported through in-depth peptide sequencing analysis, and found to carry mitochondrial DNA of the Neandertal type. Subsequent radiocarbon dating revealed the bone to be >50,000 years old. Here we demonstrate the huge potential collagen fingerprinting has for identifying hominin remains in highly fragmentary archaeological assemblages, improving the resources available for wider studies into human evolution.

Denisova Cave is a key site for our understanding of the north Asian Palaeolithic record. The cave is located near the Anui River in the Altai region of Russian Siberia (51°40′ N; 84°68′ E). Excavations undertaken by the Russian Academy of Sciences have been ongoing for more than three decades, revealing a 4.5 metre stratigraphic sequence that is key to archaeological, geological, faunal, palynological and chronological reconstructions of the Altai during the Pleistocene^1^,^2^. While this sequence has been pivotal in our understanding of Pleistocene environments, it is the hominin fossil record of the site that has become the focus of much attention. Analysis of a distal phalanx excavated from a Pleistocene level (layer 11) led to the discovery of a previously unknown hominin population, genetically distinct from both anatomically modern humans (AMH) and Neandertals^3^. In the absence of larger identifiable fossil remains that could be used to describe the holotype, the group has been named Denisovans^4^. The discovery and analysis of Neandertal remains at the site has proven equally significant. The high quality genome recently determined from a Neandertal phalanx from layer 11.4 of the East Gallery revealed that just as Neandertals and Denisovans had contributed DNA to AMHs, Neandertals had contributed DNA to Denisovans^5^. This suggests that Denisovans and Neandertals may have inhabited the Altai in close chronological proximity to one another, and even perhaps co-existed here periodically^4^,^5^. While the remains of AMHs have not yet been discovered at Denisova Cave, substantial evidence of their material culture has been found in subsequent Upper Palaeolithic layers^6^.

Hominin fossils from Denisova Cave have thus proven to be highly valuable in our understanding of archaic hominin populations. This is highlighted by the exceptional preservation state of ancient DNA molecules in some of the bones recovered at the site. The Denisovan phalanx (Denisova 3), for instance, contained >70% endogenous DNA yielding a high coverage genome (30x)^7^. Despite intensive excavation at Denisova Cave however, only a handful of hominin remains have been identified amongst the thousands of bones excavated. Those identified are either teeth or small in size, generally phalanges, which are less likely to suffer fragmentation leading to the loss of diagnostic features. Such fragmentation, which is due to both environmental taphonomy and carnivore or human activity, results in a high percentage of the bones excavated from this and many other archaeological sites which cannot be identified on the basis of their morphology^3^,^8^. Within the East Gallery of Denisova Cave alone, excavations between 2005 and 2013 yielded approximately 135,600 bones; however 128,591 could not be identified^8^.

Here we apply a method of species identification by collagen peptide mass fingerprinting, known as Zooarchaeology by Mass Spectrometry (ZooMS), to 2,315 archived unidentifiable bone fragments from Denisova Cave. These non-diagnostic bones were selected from amongst material excavated from the cave’s East Gallery in 2014. The remains varied in size, generally ranging between 3–5 cm, with bones which were large enough to be useful for additional analyses (i.e. radiocarbon and DNA analysis) preferentially selected. In the recent past, ZooMS analysis has been successful in discriminating between a diverse range of mammalian groups, including domesticated taxa^9^,^10^, wild terrestrial taxa^11^,^12^, and marine fauna^13^, as well as some non-mammalian taxa^13^,^14^. To achieve this ZooMS uses peptide mass fingerprinting to analyse the dominant protein in bone, Type 1 collagen (COL1) which is known for its longevity, particularly in cooler climates. The method has yielded collagen fingerprints in specimens dating back to ~3.5 million years^15^. COL1 is comprised of three polypeptide chains, known as alpha chains, which are constructed of a repeating pattern of amino acids. These chains carry variation in their amino acid sequence, particularly in the alpha 2 (I) chain (COL1α2), and are visible in the analysis and measurement of collagen peptides^9^. Measurement is conducted using Matrix-assisted Laser Desorption/Ionisation Mass Spectrometry (MALDI-MS), in which peptides are converted into their respective mass-to-charge (*m/z*) values. Comparison of these values with homologous values of known fauna allows for identification. A previous study^9^ proposed that seven specific collagen peptides were appropriate for zooarchaeological analysis, but with additional taxonomic groups this number has been expanded upon^13^. Although occasionally at the family/sub-family level^11^, these have been shown to most frequently identify a specimen to its genus and in some cases to species level^15^. The identification of hominin remains in this study is therefore based on the matching of previously published markers for ‘human’^9^,^12^ peptides, the majority of which could also be present in other primates due to lack of wider inclusion in these earlier studies. In the case of Denisova cave there is a very low probability of other primates being the source of hominin markers of course, due to their geographical distribution.

## Results

### ZooMS screening for hominin remains

**Figure 1:**
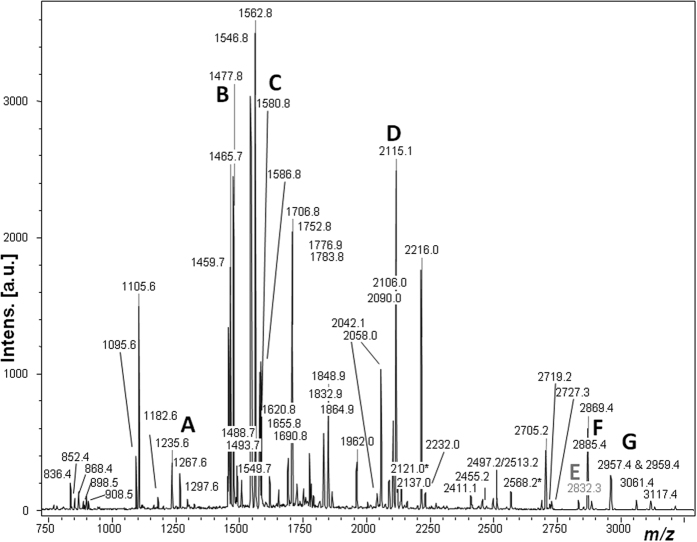
MALDI-ToF mass spectrum of digested collagen from DC1227. Previously published human markers are labelled A–G. All numbered peaks represent confirmed sequencing-matched peptides observed in human collagen (except E which is only known through similarity to homologous markers in other species^9^).

The 2,315 bones included in this study were excavated from layers 11, 12, 14 and 17 in Denisova Cave’s East Gallery. From each specimen, a bone chip ranging from 20–50 mg was removed and the bone demineralized in order to release acid-soluble proteins, which were then ultrafiltered and enzymatically digested into peptides^16^. In a single case (sample DC1227) the fingerprint identified contained all 6 of the peptide markers previously identified as human markers: *m/z* 1235.6, 1477.7, 1580.8, 2115.1, 2832.4, 2869.5 and 2957.4 (Fig. 1). However, since the original publications^9^,^12^ identifying these six markers, several have been found to appear in other taxa including afrosoricids and xenarthrans^17^. Therefore in order to confirm this peptide mass fingerprint (PMF)-based identification and the identity of the peptides present throughout the fingerprint, the sample was then analysed for peptide sequencing by LC-Orbitrap-Elite tandem mass spectrometry^18^. These analyses confirmed the dominance of human collagen in the sample (all annotated peaks in Fig. 1 were matched as *Homo* COL1A1 and COL1A2 peptides) to the exclusion of all other known mammal sequences (including other primates), identifying two new sequence PMF markers unique to Hominoidea at *m/z* 2121.0 and *m/z* 2568.2 (sequence spectra shown in Supplementary Figures S1 and S2 respectively). The LC-MS/MS data (which also yielded at least 13 non-collagenous human proteins; see Supplementary Data), specifically the combination of particular collagen peptides (e.g., the tandem spectra shown in Supplementary Figures S1–S12) was able to resolve this identification to the genus level of *Homo*, even when compared with collagen sequences from closely related primates including the other great ape genera^19^.

**Figure 2:**
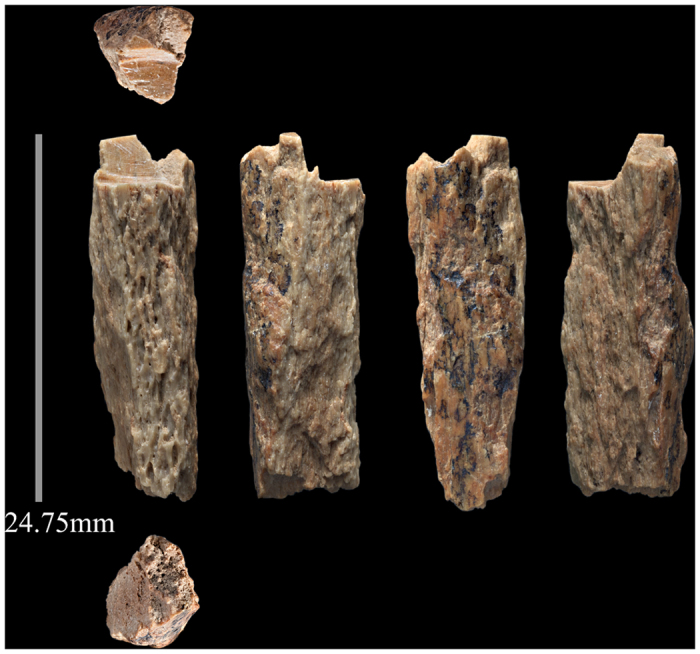
Photograph of DC1227, detailing each visible surface of the bone.

The specimen identified as originating from a hominin, DC1227, was excavated from square A-2, layer 12 of the East Gallery. Prior to sampling, the bone weighed 1.68 g, with a maximum length of 24.7 mm and width of 8.39 mm. Around 36 mg was taken for ZooMS analysis (Fig. 2). The bone appears quite unremarkable, without any morphological features or evidence for purposeful modification, it was therefore easily overlooked in osteological analysis.

### Micro CT Scan of DC1227

**Figure 3:**
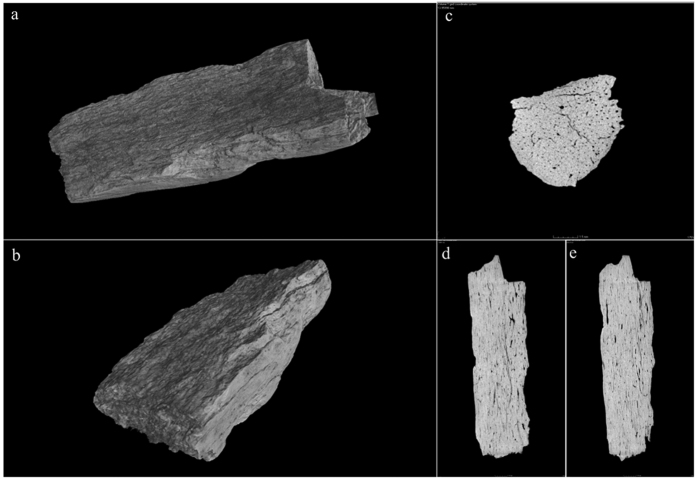
Micro-CT Scan of DC1227, (a,b) surface of DC1227, (c) projection through the length of DC1227, (d,e) projections through the width of DC1227.

Prior to further destructive sampling for radiocarbon and mitochondrial DNA analysis, micro-computed tomography (micro-CT) was performed. Given the rarity of such a discovery, a micro-CT was deemed appropriate in order to identify areas that had suffered from visible degradation and should therefore be avoided in future analysis. The results revealed the sample to be highly dense, with no signs of bone degradation despite a series of diagenetic micro-cracks running through its length. Three of these sub-micron cracks run in close proximity to one another through the bone, however they do not form a fissure and do not appear to have compromised the structure of the bone. The scan also highlighted the extent of acid etching and pitting on the bones surface, which may be the result of being passed through the digestive system of a carnivore (Fig. 3). There are a number of carnivores identified at the site; given the prevalence of hyaenas at Denisova Cave, it seems likely that the bone was subjected to acid etching via the stomach acids of a hyaena^8^,^20^.

### Radiocarbon analysis of DC1227

Prior to our work, all hominin bones discovered at the site were too small for direct radiocarbon dating, meaning that age determinations for these individuals have rested solely on their position relative to other dated materials, fauna and sediments^4^. This method assumes that the hominin remains have not been subject to post-depositional movement due to bioturbation or other taphonomic influences. This is particularly problematic when considering the limited chronological data that has so far been published for the site. Age determinations for the East Gallery are confined to a single layer (layer 11) with a series of radiocarbon dates on bones that exhibit cut marks or other human modifications, and potentially reveal some degree of mixing in that layer^4^. Radiocarbon analysis of DC1227 was therefore undertaken in order to determine that the specimen had not been reworked or intruded from higher up the sequence. While no scientific dating has so far been published for the older layer 12 of the East Gallery, studies of mammalian fauna suggest the layer is associated with species of steppe inhabitants, perhaps representing oxygen isotope stage (OIS) 4, which ended ~57,000 years ago^21^,^22^. If DC1227 was found *in situ,* it would be expected to return an age beyond the upper limit of radiocarbon dating (>50,000 years).

Radiocarbon dating was carried out at the Oxford Radiocarbon Accelerator Unit (ORAU) following standard procedures and protocols^23^ yielding an age estimate of >49,900 years BP (OxA-32241), indicating that the bone is older than the maximum measureable limit for the radiocarbon dating of bone collagen. The result is entirely consistent with its inferred geoarchaeological age with respect to the stratigraphic sequence at the site.

Isotopic measurements of carbon and nitrogen yielded a δ^13^C value of −17.3‰ and a δ^15^N value of 16.4‰. Hominins in the region typically return nitrogen isotopic values between 13–15‰, shown for example amongst the Neandertals of Okladnikov Cave^24^. The elevated values of DC1227 could indicate a variety of dietary anomalies, including a diet rich in protein derived from higher trophic level organisms such as freshwater fish^25^,^26^. Further research is required in order to place the elevated isotopic values in a proper context and this will be reported in future. Such investigations into the isotopic composition of the hominin and associated fauna from Denisova have the potential to reveal important information about the diets of Palaeolithic hominins living in the Altai and such research is currently underway at the University of Oxford.

### Mitochondrial DNA (mtDNA) sequences from specimen DC1227

We extracted DNA from 30.9 mg of bone powder from DC1227^27^. An aliquot of the extract was converted into a single-stranded DNA library^28^, which was enriched for hominin mitochondrial DNA fragments using human mitochondrial probes^29^. The isolated DNA fragments were sequenced and mapped to the revised Cambridge human mitochondrial reference sequence (rCRS). In total, we identified 282,502 unique mtDNA fragments longer than 35 base pairs (Supplementary Table S1).

To assess whether some of the mtDNA fragments were of ancient origin, we made use of the fact that cytosine (C) bases at the ends of DNA molecules over time tend to undergo deamination^30^ and as a result are read as thymines (T) by DNA polymerases. Ancient DNA fragments aligned to a reference sequence thus tend to carry high frequencies of apparent C to T substitutions at their 5′- and 3′- ends^31^,^32^,^33^. Of the fragments starting or ending at positions where the rCRS base is a C, 32.2% and 31.3% carried Ts at their 5′- and 3′- ends, respectively (Supplementary Figure S13), indicating that ancient hominin DNA molecules are present in DC1227.

To determine whether the endogenous mtDNA of DC1227 is most closely related to modern human, Neandertal or Denisovan mitochondrial genomes, we restricted the analysis to sequences carrying a C to T substitution relative to the rCRS at one of their ends^34^ to diminish the influence of putative contamination by present-day human DNA (Supplementary Information). Using 36,665 such sequences (Supplementary Table S1), the mitochondrial genome of specimen DC1227 was reconstructed with an average coverage of 130-fold, leaving 63 positions covered by two or fewer sequences and four where fewer than two thirds of sequences carried the same base. Thus, 67 positions could not be confidently called.

**Figure 4:**
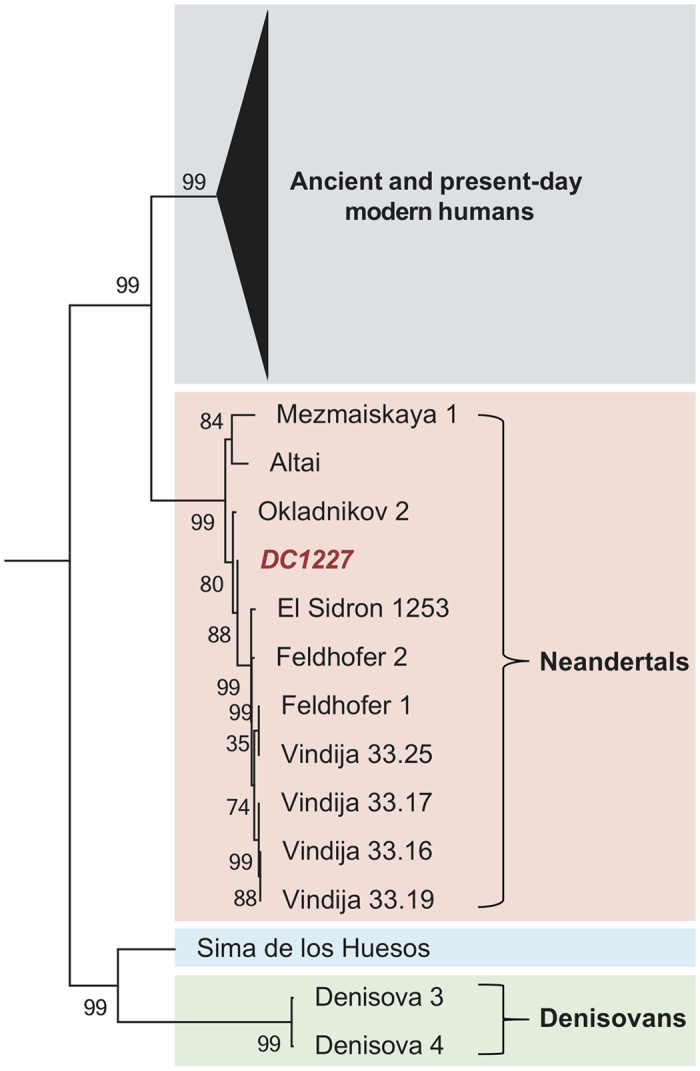
Neighbor-joining tree relating the DC1227 mtDNA to other ancient and present-day mtDNAs. A chimpanzee mtDNA was used as an outgroup (not shown). Support for each branch is based on 500 bootstrap replications. See Table S2 for the geographic origin of the ancient specimens.

When comparing the DC1227 mtDNA to complete Neandertal mtDNA determined to date, it carries five base differences to the Neandertal mtDNA of Okladnikov 2^33^ found approximately 60 km from Denisova Cave, 12 to 17 differences to Neandertals from western and southern Europe, and 31 differences to Mezmaiskaya 1 from the Caucasus^35^ and to a Neandertal found in Denisova Cave^5^. In comparison, the mtDNA of DC1227 differs by between 174 and 354 bases to the mtDNAs of other hominin groups (Supplementary Table S2). In a phylogenetic analysis (Fig. 4), the DC1227 mitochondrial genome thus falls within the variation of the ten Neandertals, to the exclusion of 311 present-day humans, ten ancient modern humans, two Denisovans, and a Middle Pleistocene hominin from Spain. We conclude that the DC1227 individual carried a mitochondrial genome of the Neandertal type and refer to it henceforth as Denisova 11.

## Conclusions

The crucial importance of genetic analysis for palaeoanthropology is apparent in the insights that have been made in the recent years. The dearth of human skeletal material from Pleistocene-age deposits constitutes a major drawback for future applications of these methods. By utilising assemblages of bone already excavated from archaeological sites it may be possible to identify hominin remains useful for a variety of scientific investigations. Collagen fingerprinting using ZooMS is highly effective at identifying such material for further scientific investigation and it is this kind of multi-disciplinary approach that holds the key to broadening our understanding of Pleistocene human populations. At Denisova Cave we have shown the feasibility of successfully carrying out such research. Here, and in the wider Altai region, there is the additional possibility of attaining high genetic coverage due to the favourable climatic and post-depositional conditions. Ongoing work is now focusing on screening additional material from this important site in an attempt to identify more hominin remains. The rapid nature of ZooMS, and its amenability to high-throughput methodology, has the potential to identify undiagnostic and fragmented hominin remains at key sites. This is the first time a novel combination of ZooMS, radiocarbon dating and ancient DNA analysis have been performed in order to identify and analyse human fragments from an archaeological site. Increased utility of this combined methodology could ultimately provide the source material necessary to further our understanding of human evolution.

## Methods

### ZooMS

Between 20–50 mg of bone were taken from each of the bones within the assemblage and demineralised in 0.6 M hydrochloric acid (HCl) for 18 hours. The resulting residue was removed into 30 kDa molecular weight cut-off (MWCO) ultrafilters and centrifuged at 3700 rpm for 1 hour. The filtrate was then twice washed through with 500 μL of 50 mM ammonium bicarbonate (AmBic) and further centrifuged at 3700 rpm for half an hour after each treatment. The final residue was resuspended with additional AmBic (200 μL), half of which was removed to create a backup sample set before digestion. The remaining 100 μL was then treated with 0.2 μg trypsin (sequencing grade; Promega UK) and incubated at 37 °C for 18 hours. The resulting solution was then mixed with a matrix solution of 1 μl of α-cyano-4-hydroxycinnamic acid solution (10 mg/mL in 50% acetonitrile (ACN)/0.1% trifluoroacetic acid (TFA)), allowed to co-crystalise and analysed using a Bruker Ultraflex II (Bruker Daltonics, Bremen) MALDI Tof/Tof mass spectrometer. The resulting mass spectra were screened for the human markers^9^,^12^ within FlexAnalysis software.

For peptide sequencing, the pellet following demineralisation was further extracted for 18 h at 4 °C in a buffer containing 100 mM Tris and 6 M GuHCl at pH 7.4 and then ultrafiltered into 50 mM AmBic as described above. Following reduction and alkylation, the proteins were digested with trypsin as above and then acidified to 0.1% TFA and desalted, purified and concentrated with 100 μL C18 reversed-phase Zip-Tips (Millpore)^18^, eluting with 50% ACN/0.1% TFA. Following purification the sample was lyophilised by evaporation and resuspended in 20 μL 0.1% TFA. The sample peptides were then analysed by LC/MS/MS using an UltiMate® 3000 Rapid Separation LC (RSLC, Dionex Corporation, Sunnyvale, CA, USA) coupled to an Orbitrap Elite (Thermo Fisher Scientific, Waltham, MA, USA) mass spectrometer (120 k resolution, full scan, positive mode, normal mass range 350–1500). Peptides were separated using an Ethylene Bridged Hybrid (BEH) C18 analytical column (75 mm × 250 μm i.d., 1.7 μM; Waters) with a gradient from 92% A (0.1% formic acid in water) and 8% B (0.1% formic acid in acetonitrile) to 33% B in 44 min at a flow rate of 300 nL min^–1^ and automatically selected for fragmentation by data-dependent analysis; six MS/MS scans (Velos ion trap, product ion scans, rapid scan rate, Centroid data; scan event: 500 count minimum signal threshold, top 6) were acquired per cycle, dynamic exclusion was employed, and 1 repeat scan (i.e. two MS/MS scans total) was acquired in a 30 s repeat duration with that precursor being excluded for the subsequent 30 s (activation: collision-induced dissociation (CID), 2+ default charge state, 2 *m/z* isolation width, 35 eV normalized collision energy, 0.25 Activation Q, 10.0 ms activation time). The data consisting of 11,651 peptide ions were searched against the SwissProt database (www.ebi.ac.uk/swissprot/) for matches to primary protein sequences using the Mascot search engine (version 2.5.1; Matrix Science, London, UK) including the fixed carbamidomethyl modification of cysteine and the variable modifications for deamidation, and oxidation of lysine, proline and methionine residues to account for common PTMs and diagenetic alterations. Enzyme specificity was limited to trypsin/P with up to 2 missed cleavages allowed, mass tolerances were set at 5 ppm for the precursor ions and 0.5 Daltons for the fragment ions, all spectra were considered as having either 2+ or 3+ precursors and the peptide ion score cut off was set at 42 as identified by the initial Mascot output.

### CT Scanning

The CT scan was undertaken using a Nikon XT H 225 micro-scanner with a transmission target. Attempts to keep the dosage as low as possible were made in order to avoid any damage to the sample, so the scan was run at 70kv and 80 μA. In total 1,448 projects were taken at two frames per projection, with an exposure set at 100ms and magnification at ×7.2. Data was reconstructed using CT Pro 3D software, and processed with VG Studio Max 2.1 software.

### Mitochondrial DNA Analysis

#### DNA extraction and library preparation

30.9 mg of bone powder was removed from DC1227 using a dentistry drill. DNA was extracted using a silica-based protocol designed to retrieve short DNA molecules^27^,^36^. 10 μl of the DNA extract (E3128) were converted into a single-stranded DNA library (A9301), as described^28^,^36^. The number of DNA molecules in the library was assessed by digital droplet PCR (BioRad QX 200), using 1 μl as input for an EvaGreen (BioRad) assay with primers IS7 and IS8^37^. The library was barcoded with two unique indexes^36^,^38^ and amplified using AccuPrime Pfx DNA polymerase (Life Technologies)^39^. Amplification products were purified using the MinElute PCR purification kit (Qiagen); and quantified on a NanoDrop ND-1000 (NanoDrop Technologies) photospectrometer.

#### Mitochondrial capture and sequencing

The amplified library (A9317) was enriched by a bead-based hybridization capture protocol^29^ using 52-mer probes^40^ designed in single base pair tiling on the rCRS (National Center for Biotechnology Information [NCBI] reference NC_012920) in two rounds of capture, using 1 μg and 0.5 μg of input DNA, respectively. The captured library (L5502) was sequenced on a MiSeq (Illumina) platform, using paired-end runs (2 × 76 cycles) with double-index configuration^38^. One DNA extraction blank and one library preparation blank were carried along the entire procedure as negative controls.

#### Sequence processing and mapping

Base calling was performed using Bustard (Illumina). Adapter sequences were trimmed, and forward and reverse reads were merged into single sequences^41^. Sequences lacking perfect matches to the expected barcodes were discarded. Mapping to the reference genome was carried out using BWA^42^, with parameters “-n 0.01 -o 2 -l 16500” 7. PCR duplicates were removed by merging sequences with identical alignment start and end coordinates, using bam-rmdup (https://github.com/udo-stenzel/biohazard). Sequences longer than 35 bases with a mapping quality greater than 30 were retained for further analyses.

#### Phylogenetic analysis

Sequences carrying a terminal C to T substitution were used to reconstruct the DC1227 mtDNA. Terminal Ts at the first or last positions of each sequence were converted to Ns to reduce the impact of damage-derived sequence errors in consensus calling. A consensus base was determined if a position was covered by at least three sequences, and if at least 67% of sequences, *i.e.* more than two thirds overlapping it carried an identical base^34^.

The mtDNA was aligned to the mtDNAs of 311 present-day humans^43^, 10 ancient modern humans^31^,^44^,^45^,^46^,^47^ ten Neandertals^5^,^33^,^35^,^48^,^49^, two Denisovans^3^,^4^ a Middle Pleistocene hominin^34^ and a chimpanzee (*Pan troglodytes*, NC_001643)^50^ by MAFFT^51^. The number of base differences between sequences and a Neighbor-Joining tree with 500 bootstrap replications^52^ based on these differences were produced using MEGA6^53^.

## Additional Information

**How to cite this article**: Brown, S. *et al.* Identification of a new hominin bone from Denisova Cave, Siberia using collagen fingerprinting and mitochondrial DNA analysis. *Sci. Rep.* **6**, 23559; doi: 10.1038/srep23559 (2016).

**Accession code**: The mitochondrial genome sequence of specimen DC1227 (Denisova 11) was deposited in GenBank (accession number KU131206).

## Supplementary Material

Supplementary Information

Supplementary Data 1
