# Supplementary material for: Identification of a new hominin bone from Denisova Cave, Siberia using collagen fingerprinting and mitochondrial DNA analysis
Source: Sci Rep. 2016 Mar 29;6:23559. doi: 10.1038/srep23559 (PMC4810434; doi:10.1038/srep23559)
Supplement: Supplementary Information [file srep23559-s1.pdf]

Identification of a new hominin bone from Denisova Cave, Siberia using collagen fingerprinting and mitochondrial DNA analysis

Samantha Brown[1] *, Thomas Higham[1] *, Viviane Slon[2], Svante Pääbo[2], Matthias Meyer[2], Katerina Douka[1], Fiona Brock[3], Daniel Comeskey[1], Noemi Procopio[4], Michael Shunkov[5], Anatoly Derevianko[5], Michael Buckley[4] *

[1]RLAHA, University of Oxford, OX1 3QY, UK

[2]MPI-EVA, Leipzig, 04103,Germany

[3]Cranfield Forensic Institute, Cranfield University, SN6 8LA, UK

[4]Faculty of Life Sciences, University of Manchester, M13 9PL, UK

[5]Institute of Archeology and Ethnography, Novosibirsk, 630090, Russia

*corresponding authors can be contacted via samantha.brown@rlaha.ox.ac.uk; thomas.higham@rlaha.ox.ac.uk; m.buckley@manchester.ac.uk

Supplementary Information

ZooMS Analysis

MS/MS Fragmentation of **GLHGEFGLPGPAGPR**
Found in **CO1A2_HUMAN** in **SwissProt**, Collagen alpha-2(I) chain OS=Homo sapiens GN=COL1A2 PE=1 SV=7

Match to Query 3029: 1476.748754 from(739.381653,2+) index(8121)
Title: 20150703_MB2_Stand.6040.6040.2.dta
Data file 20150703_MB2_Stand.raw.mgf

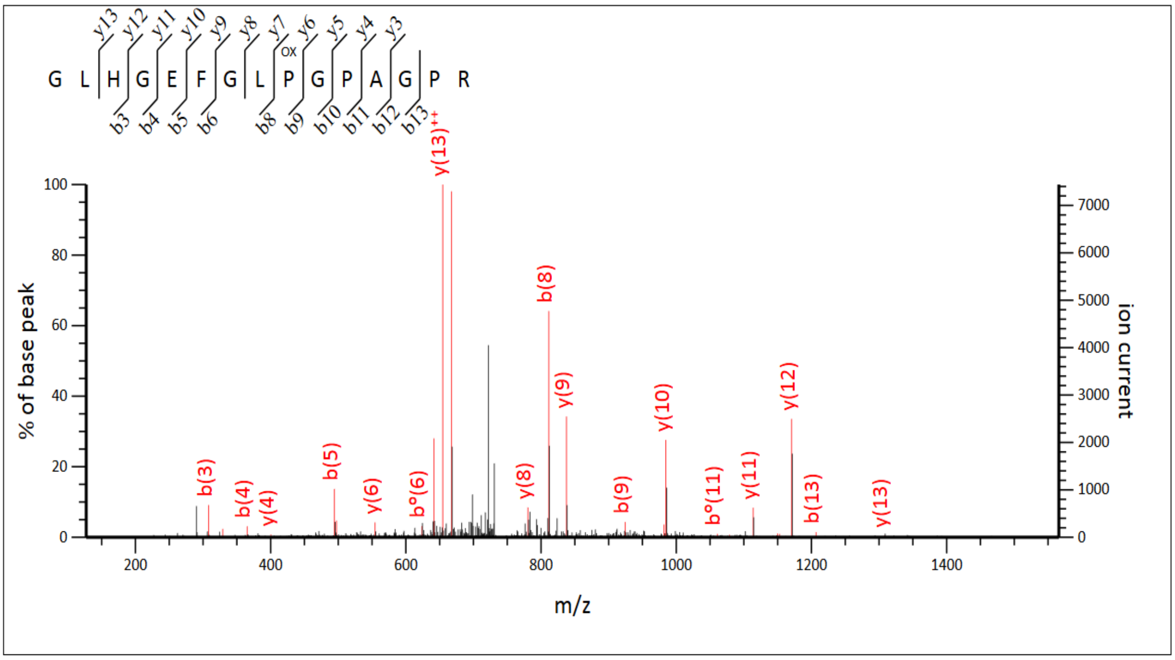

Supplementary Figure S1. Tandem mass spectrum of peak at m/z 1477.7 representing peptide sequence GLHGEFGLPGPAGPR

MS/MS Fragmentation of **GEIGAVGNAGPAGPAGPR**
Found in **CO1A2_HUMAN** in **SwissProt**, Collagen alpha-2(I) chain OS=Homo sapiens GN=COL1A2 PE=1 SV=7

Match to Query 3472: 1547.770364 from(774.892458,2+) index(7195)
Title: 20150703_MB2_Stand.5029.5029.2.dta
Data file 20150703_MB2_Stand.raw.mgf

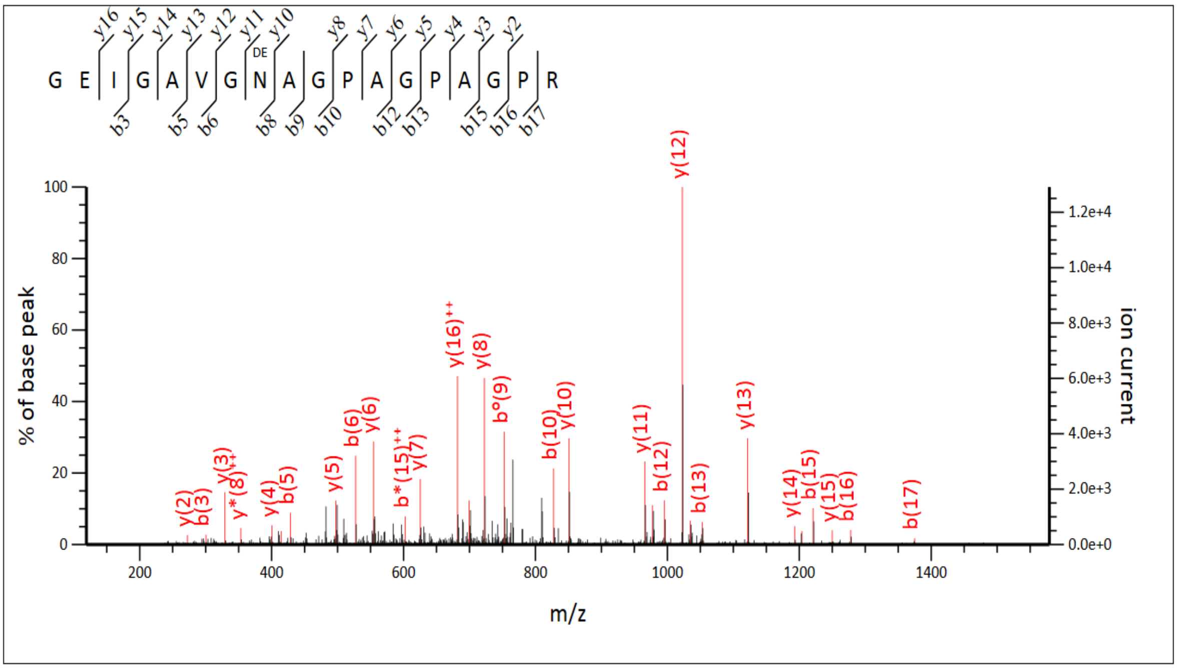

Supplementary Figure S2. Tandem mass spectrum of peak at m/z 1548.8 representing peptide sequence GEIGAVGNAGPAGPAGPR

MS/MS Fragmentation of **GPNGEAGSAGPPGPPGLR**
Found in **CO1A2_HUMAN** in **SwissProt**, Collagen alpha-2(I) chain OS=Homo sapiens GN=COL1A2 PE=1 SV=7

Match to Query 4000: 1618.769264 from(810.391908,2+) index(6221)
Title: 20150703_MB2_Stand.3930.3930.2.dta
Data file 20150703_MB2_Stand.raw.mgf

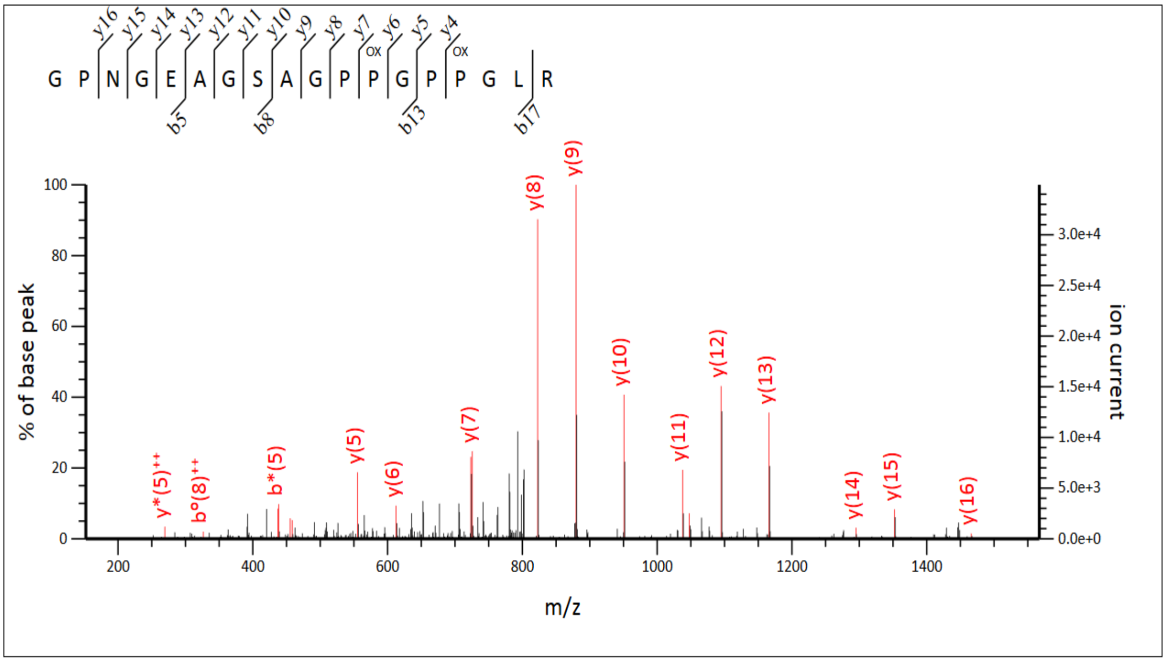

Supplementary Figure S3. Tandem mass spectrum of peak at m/z 1619.8 representing peptide sequence GPNGEAGSAGPPGPPGLR

MS/MS Fragmentation of **GLPGSPGNIGPAGK**
Found in **CO1A2_HUMAN** in **SwissProt**, Collagen alpha-2(I) chain OS=Homo sapiens GN=COL1A2 PE=1 SV=7

Match to Query 1953: 1252.641694 from(627.328123,2+) index(5936)
Title: 20150703_MB2_Stand.3601.3601.2.dta
Data file 20150703_MB2_Stand.raw.mgf

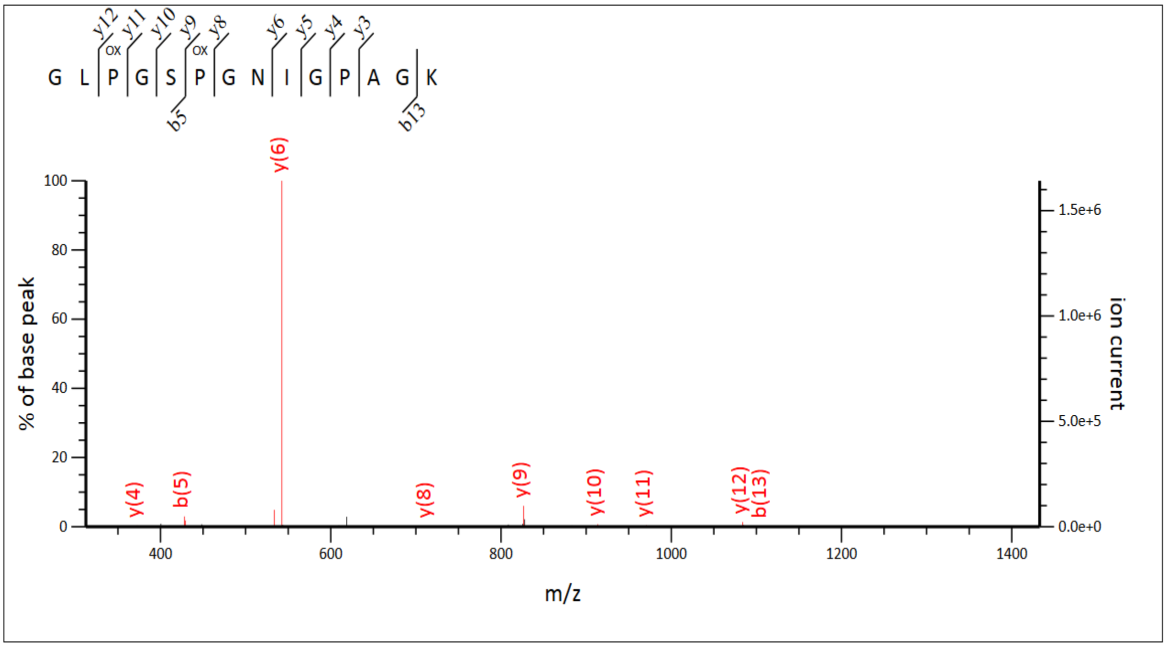

Supplementary Figure S4. Tandem mass spectrum of peak at m/z 1253.6 representing peptide sequence GLPGSPGNIGPAGK

MS/MS Fragmentation of **GEPGVVGAVGTAGPSGPSGLPGER**
Found in **CO1A2_HUMAN** in **SwissProt**, Collagen alpha-2(I) chain OS=Homo sapiens GN=COL1A2 PE=1 SV=7

Match to Query 6706: 2120.051614 from(1061.033083,2+) index(9093)
Title: 20150703_MB2_Stand.7101.7101.2.dta
Data file 20150703_MB2_Stand.raw.mgf

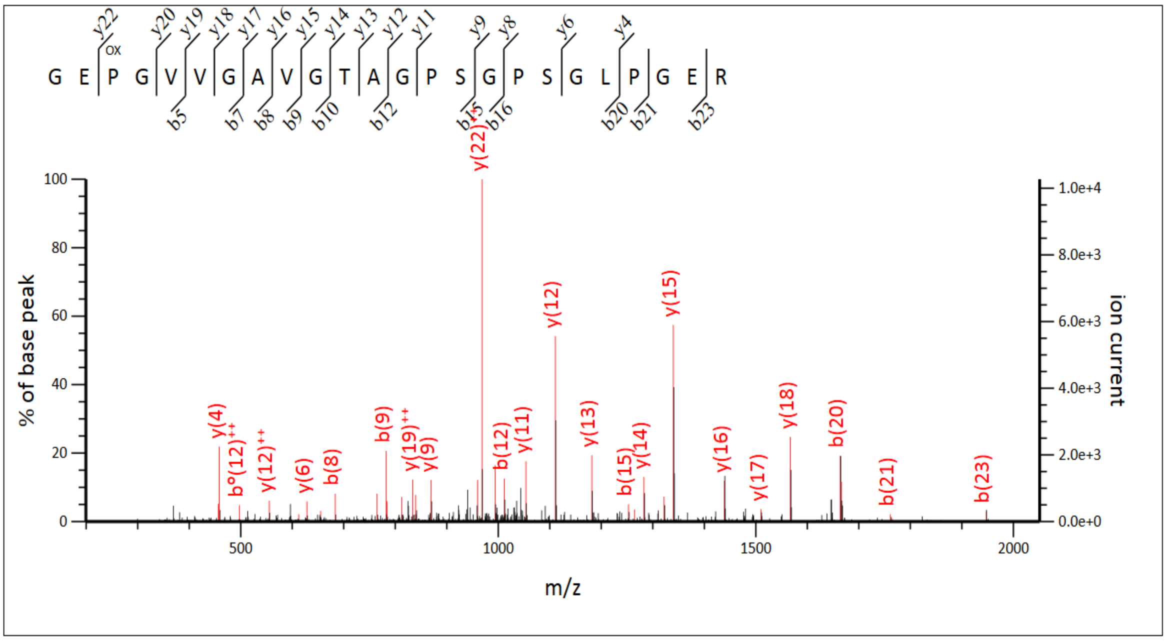

Supplementary Figure S5. Tandem mass spectrum of peak at m/z 2121.1 representing peptide sequence GEPGVVGAVGTAGPSGPSGLPGER

MS/MS Fragmentation of **GENGVVGPTGPVGAAGPAGPNGPPGPAGSR**
Found in **CO1A2_HUMAN** in **SwissProt**, Collagen alpha-2(I) chain OS=Homo sapiens GN=COL1A2 PE=1 SV=7

Match to Query 8793: 2567.243014 from(1284.628783,2+) index(8328)
Title: 20150703_MB2_Stand.6269.6269.2.dta
Data file 20150703_MB2_Stand.raw.mgf

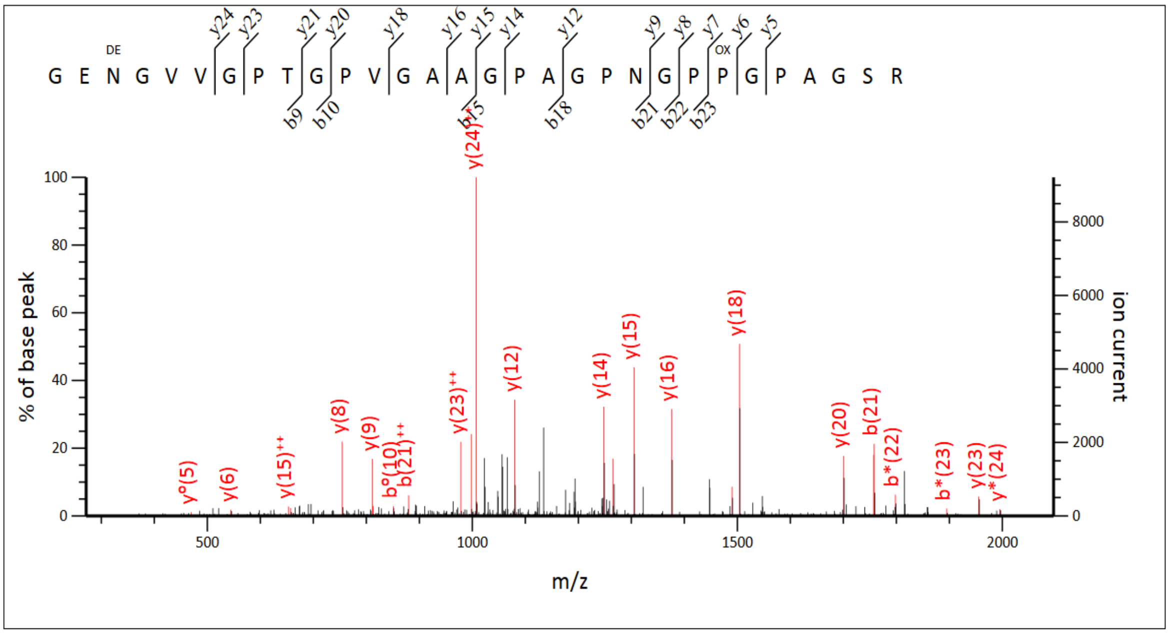

Supplementary Figure S6. Tandem mass spectrum of peak at m/z 1285.6 representing peptide sequence GENGVVGPTGPVGAAGPAGPNGPPGPAGSR

MS/MS Fragmentation of **TGEVGAVGPPGFAGEK**
Found in **CO1A2_HUMAN** in **SwissProt**, Collagen alpha-2(I) chain OS=Homo sapiens GN=COL1A2 PE=1 SV=7

Match to Query 3116: 1487.728854 from(744.871703,2+) index(7607)
Title: 20150703_MB2_Stand.5484.5484.2.dta
Data file 20150703_MB2_Stand.raw.mgf

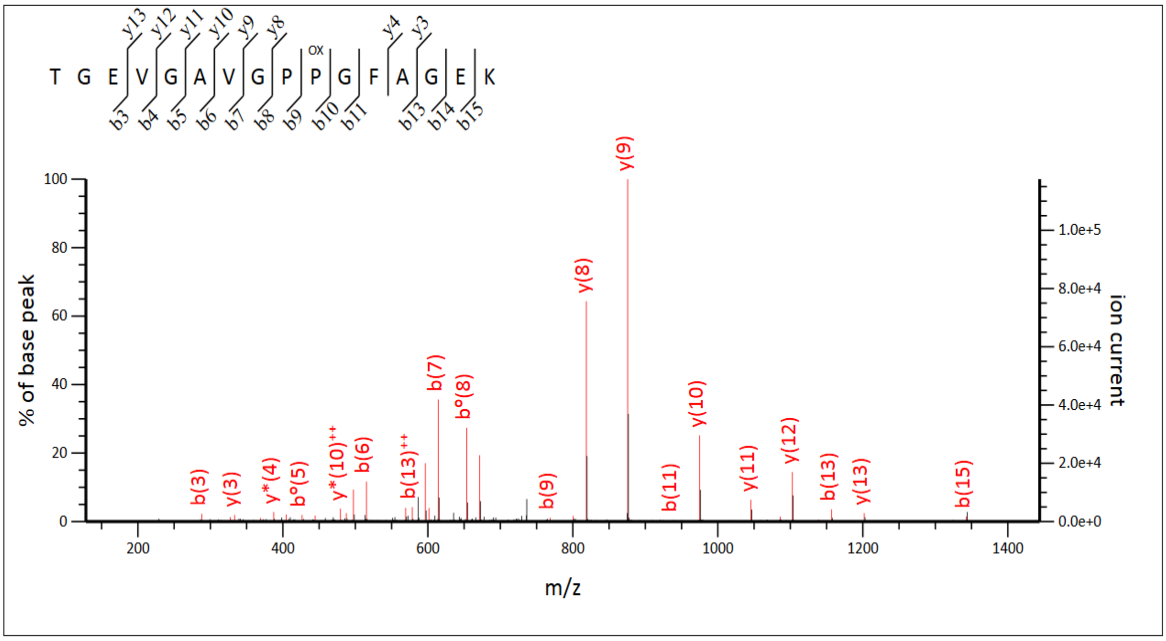

Supplementary Figure S7. Tandem mass spectrum of peak at m/z 1488.7 representing peptide sequence TGEVGAVGPPGFAGEK

MS/MS Fragmentation of **GPSGEAGTAGPPGTPGPQGLLGAPGILGLPGSR**
Found in **CO1A2_HUMAN** in **SwissProt**, Collagen alpha-2(I) chain OS=Homo sapiens GN=COL1A2 PE=1 SV=7

Match to Query 10169: 2956.493504 from(1479.254028,2+) index(785)
Title: 20150703_MB2_Stand.10976.10976.2.dta
Data file 20150703_MB2_Stand.raw.mgf

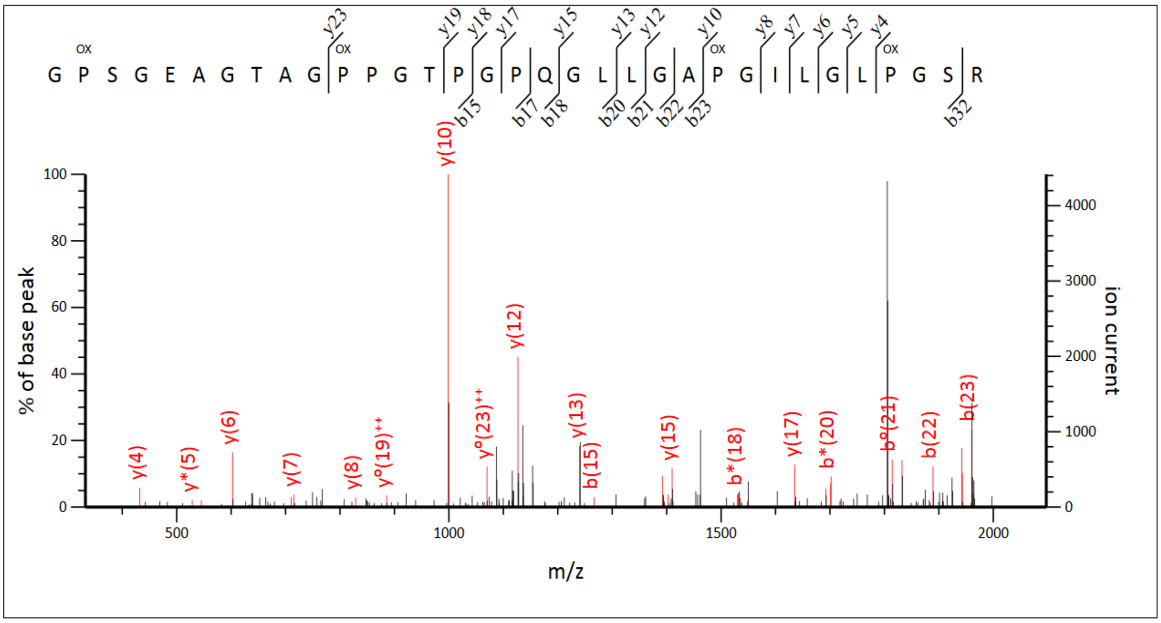

Supplementary Figure S8. Tandem mass spectrum of peak at m/z 2957.5 representing peptide sequence GPSGEAGTAGPPGTPGPQGLLGAPGILGLPGSR

MS/MS Fragmentation of **GLPGVAGAVGEPGPLGIAGPPGAR**
Found in **CO1A2_HUMAN** in **SwissProt**, Collagen alpha-2(I) chain OS=Homo sapiens GN=COL1A2 PE=1 SV=7

Match to Query 6786: 2130.112644 from(1066.063598,2+) index(10303)
Title: 20150703_MB2_Stand.8438.8438.2.dta
Data file 20150703_MB2_Stand.raw.mgf

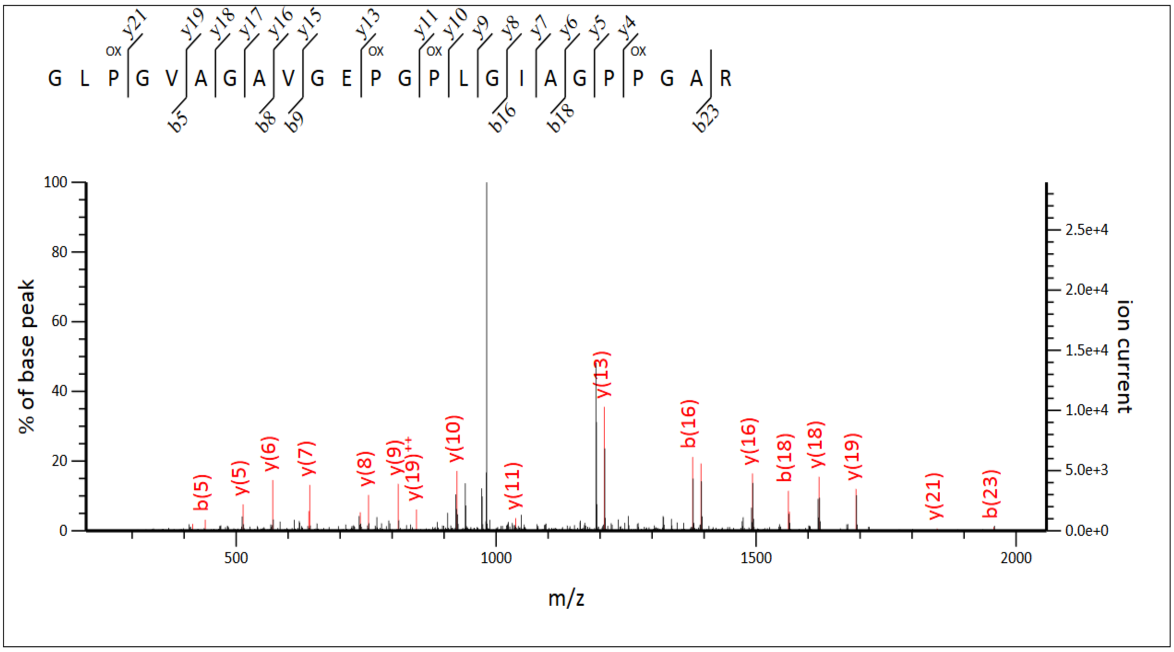

Supplementary Figure S9. Tandem mass spectrum of peak at m/z 2131.1 representing peptide sequence GLPGVAGAVGEPGPLGIAGPPGAR

MS/MS Fragmentation of **GYPGNIGPVGAAGAPGPHGPVGPAGK**
Found in **CO1A2_HUMAN** in **SwissProt**, Collagen alpha-2(I) chain OS=Homo sapiens GN=COL1A2 PE=1 SV=7

Match to Query 7591: 2284.129244 from(1143.071898,2+) index(7627)
Title: 20150703_MB2_Stand.5505.5505.2.dta
Data file 20150703_MB2_Stand.raw.mgf

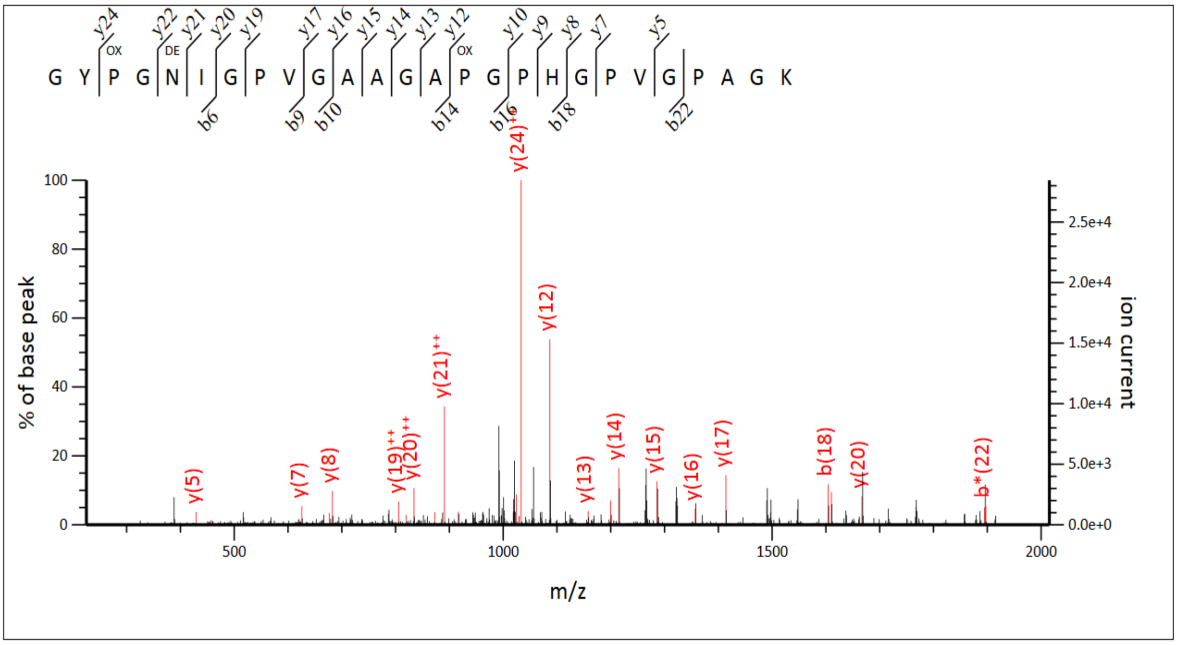

Supplementary Figure S10. Tandem mass spectrum of peak at m/z 2285.1 representing peptide sequence GYPGNIGPVGAAGAPGPHGPVGPAGK

MS/MS Fragmentation of **GETGPSGPVGPAGAVGPR**
Found in **CO1A2_HUMAN** in **SwissProt**, Collagen alpha-2(I) chain OS=Homo sapiens GN=COL1A2 PE=1 SV=7

Match to Query 3561: 1561.788914 from(781.901733,2+) index(6856)
Title: 20150703_MB2_Stand.4658.4658.2.dta
Data file 20150703_MB2_Stand.raw.mgf

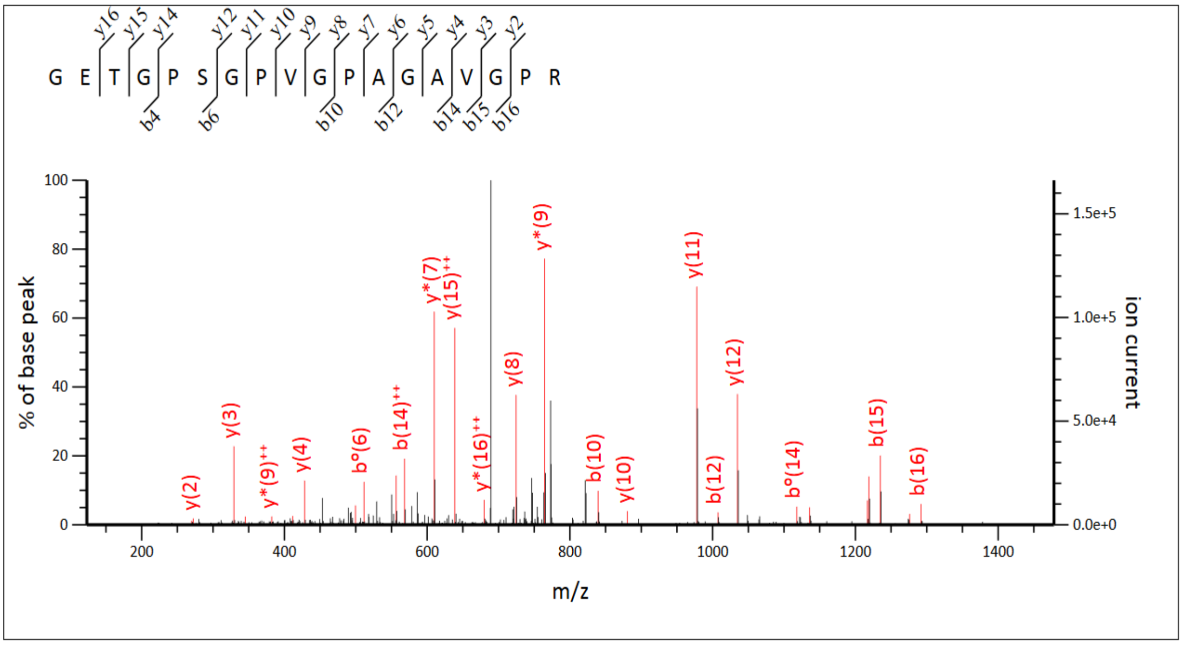

Supplementary Figure S11. Tandem mass spectrum of peak at m/z 1562.8 representing peptide sequence GETGPSGPVGPAGAVGPR

MS/MS Fragmentation of **AGVMGPPGSR**
Found in **CO1A2_HUMAN** in **SwissProt**, Collagen alpha-2(I) chain OS=Homo sapiens GN=COL1A2 PE=1 SV=7

Match to Query 770: 959.449554 from(480.732053,2+) index(1473)
Title: 20150703_MB2_Stand.1182.1182.2.dta
Data file 20150703_MB2_Stand.raw.mgf

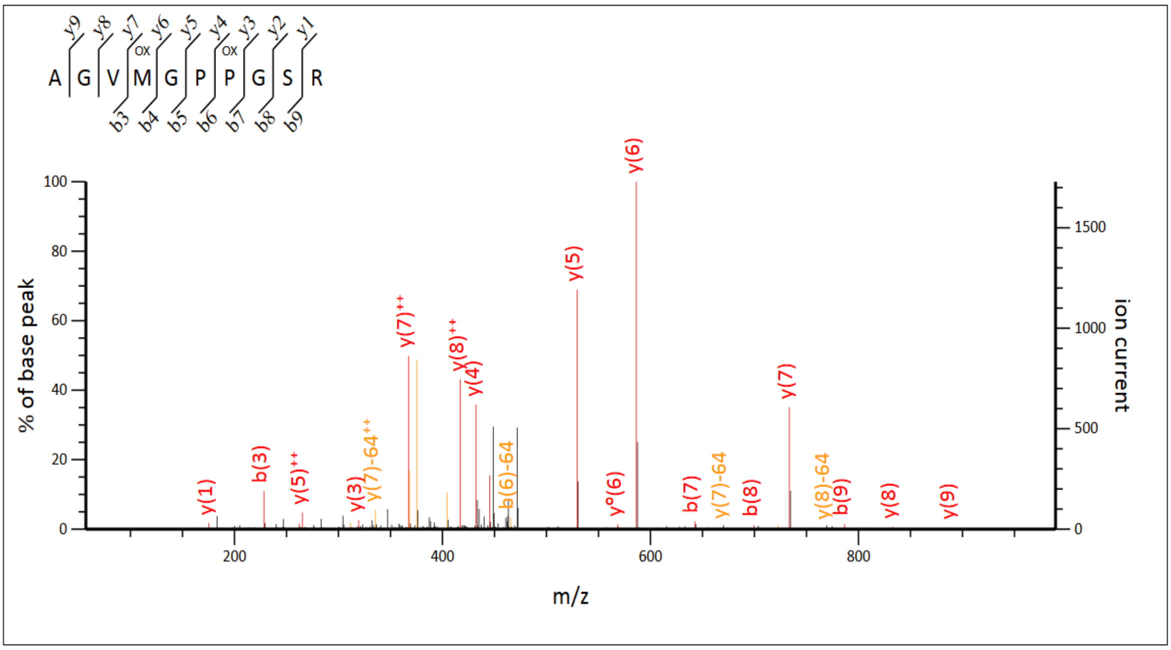

Supplementary Figure S12. Tandem mass spectrum of peak at m/z 960.5 representing peptide sequence AGVMGPPGSR

Mitochondrial DNA Analysis

Contamination estimate

To estimate the amount of contamination by present-day modern human DNA in library L5502, we used 72 positions at which the DC1227 mtDNA differs from 311 present-day human mtDNAs (Green et al., 2010). Out of 30,705 DNA fragments overlapping these positions, 2,301 carried present-day human bases, indicating a contamination of 7.49%. Among the fragments with apparent C to T substitution at their 5'- or 3'-ends, 0.94% (27 out of 2,865) carried present-day human bases.

Supplementary Table S1. Characteristics of the DNA libraries captured with human mitochondrial probes.

Library ID	Sample ID	Material used for extraction [mg]	Molecules in library (ddPCR estimate)	Number of raw sequences	Number of aligned sequences	Sequences mapped [%]	Number of unique sequences	Average number of duplicates	Number of sequences with C to T substitution
L5502	DC1227	30.9	3.96E+09	2,303,491	1,457,145	71.899	282,502	5.16	36,665
L5506	ENC	-	6.50E+07	749,443	131,316	74.855	698	188.13	42
L5507	LNC	-	4.55E+07	351,160	138,265	84.344	385	359.13	19

ddPCR – digital droplet PCR; C – cytosine; T – thymine; ENC – extraction negative control; LNC – library preparation negative control

Supplementary Table S2. Number of base substitutions between the DC1227 mtDNA and other mtDNAs. Note that 67 positions in the mitochondrial genome of DC1227 were not called. For 311 present-day humans, the range and average number of differences are reported. The geographical origin of each specimen is noted in parentheses.

Sample ID (geographical origin)	Number of differences to DC1227	Sample ID (geographical origin)	Number of differences to DC1227
**Neandertals**		**Ancient modern humans**	
Okladnikov 2 (Siberia) [a]	5	Boshan 11 (China) [i]	176
Feldhofer 2 (Germany) [b]	12	Ust Ishim (Siberia) [j]	177
El Sidron 1253 (Spain) [b]	13	Tianyuan (China) [k]	178
Vindija 33.17 (Croatia) [c]	15	Dolni Vestonice 14 (Czech Republic) [i]	179
Feldhofer 1 (Germany) [b]	15	Kostenki (Russia) [l]	180
Vindija 33.25 (Croatia) [b]	15	Saqqaq (Greenland) [m]	180
Vindija 33.16 (Croatia) [d]	17	Oberkassel 998 (Germany) [i]	183
Vindija 33.19 (Croatia) [c]	17	Dolni Vestonice 13 (Czech Republic) [i]	184
Mezmaiskaya 1 (Caucasus) [b]	31	Loschbour (Luxembourg) [i]	185
Altai (Siberia) * [e]	31	Iceman (Austro-Italian border) [n]	185
**Denisovans**		**Present-day modern humans**	
Denisova 3 (Siberia) * [f]	354	Average (world-wide) [o]	185
Denisova 4 (Siberia) * [g]	354	Range	174-202
**Sima de los Huesos hominin**			
Sima de los Huesos (Spain) [h]	270		

* indicates that the specimen was found in Denisova Cave, as is the case for DC1227.
Comparative sequence data was taken from [a] Skoglund et al., 2014; [b] Briggs et al., 2009; [c] Gansauge & Meyer, 2014; [d] Green et al., 2008; [e] Prüfer et al., 2014; [f] Krause et al., 2010B; [g] Reich et al., 2010; [h] Meyer et al., 2014; [i] Fu et al., 2013B; [j] Fu et al., 2014; [k] Fu et al., 2013A; [l] Krause et al., 2010A; [m] Gilbert et al., 2008; [n] Ermini et al., 2008; [o] Green et al., 2010.

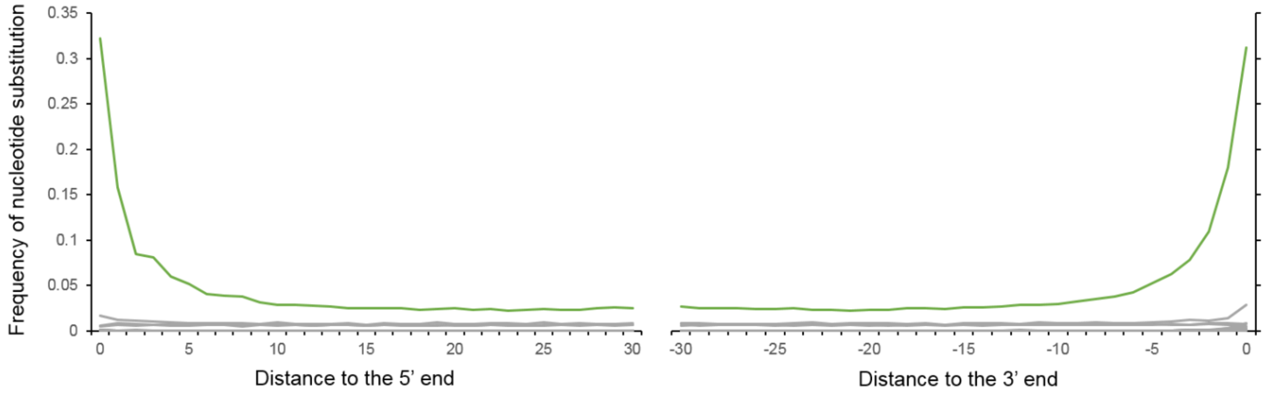

Supplementary Figure S13. Frequencies of nucleotide substitutions towards each end of sequences retrieved from DC1227. C to T substitutions are marked in green, all other types of nucleotide changes in gray.
